# A possible role of lncRNA MEG3 and lncRNA MAFG-AS1 on miRNA 147-b in the pathogenesis of Behcet’s disease

**DOI:** 10.1007/s00251-024-01346-8

**Published:** 2024-07-10

**Authors:** Shimaa Abobakr, Olfat Shaker, Mohamed Tharwat Hegazy, Ayman Mohamed Hany

**Affiliations:** 1 Medical Biochemistry and Molecular Biology, Faculty of Medicine, Cairo University, Cairo, Egypt; 2 Internal Medicine Department, Rheumatology and Clinical Immunology Unit, Faculty of Medicine, Cairo University, Cairo, Egypt

**Keywords:** Behcet’s disease, lncRNA MEG3, lncRNA MAFG-AS1, miRNA147-b

## Abstract

Behcet’s disease (BD) is a multisystem disease with altered Toll-like receptors (TLRs) on macrophages. Long noncoding RNA Maternally expressed gene 3 (lncRNA MEG3) and lncRNA Musculoaponeurotic fibrosarcoma oncogene family, protein G antisense 1 (MAFG-AS1) are regulators of microRNA (miRNA) 147-b, which is induced upon TLR stimulation. We included fifty BD patients, and fifty age and sex-matched controls. Real-time polymerase chain reaction (PCR) was used to measure the expression levels of serum lncRNA MEG3, lncRNA MAFG-AS1, and miRNA 147-b. LncRNA MEG3 and lncRNA MAFG-AS1 were significantly downregulated while miRNA 147-b was significantly upregulated in the BD patients' serum compared to the controls with *p*-value <0.001. Receiver operation characteristics (ROC) curve analysis revealed that the three biomarkers can discriminate between BD and control subjects with 76%, 100%, and 70% sensitivity respectively, and 100% specificity for all of them. There was a lower expression level of lnc RNA MEG3 among patients who had new eye involvement in the last month in comparison to those without new eye involvement (*p*-value=0.017). So, LncRNA MEG3, lncRNA MAFG-AS1, and miRNA147-b are promising diagnostic markers and therapeutic targets for BD patients. LncRNA MEG3 can be used as a predictor for new BD ocular involvement.

## Introduction

Behcet’s disease (BD) is a multisystemic disease affecting many organs in the body especially skin, mucocutaneous membranes, eyes, and vessels (Bettiol et al. 2023). Notably, 50–70% of BD patients experience ophthalmic manifestations, which can end in blindness in 25% of the affected patients within 10 years (Posarelli et al. 2020). If the patient’s disease activity is not well controlled, dramatic sequelae will occur which affects the patient’s health and in turn his community. The disease outcomes may result in major disabilities in young age patients (Atas et al. 2019).

While BD pathogenesis has been described by many theories, these theories are still not enough to fully understand this disease. In general, it is described as having an aberrant innate hyperinflammation associated with neutrophil hyperactivity (Perazzio et al. 2020). One of the theories described an altered Toll-like receptor (TLR) expression on the surface of the macrophages and neutrophils that contributes to excessive innate immune activation in BD (Van der Houwen et al. 2020). Moreover, NF-KB (Nuclear Factor Kappa B) hyperactivation has been mentioned as a participant inflammatory condition in BD (Perazzio et al. 2020).

Nearly 70% of human genes are transcribed as noncoding ribonucleic acids (ncRNAs). Long noncoding RNAs (lncRNAs) and micro RNAs (miRNAs) are two main categories of them (Wang 2023). LncRNAs are newly recognized ncRNAs measuring 200 nucleotides (NTs) or more and they were proven to be involved in innate immune response and inflammation, and they have been linked with the development of numerous autoimmune and inflammatory diseases. Their functions are mediated by different mechanisms, one is sponging miRNAs to restrain messenger RNA degradation and translation repression (Xie and Wei 2021).

MiRNAs are ncRNAs measuring nearly 22 NTs. They are considered essential regulators of T and B immune cell development and functions. Also, they were proven to play significant functions in different human immune disorders like rheumatoid arthritis, systemic lupus erythematosus (Zhang et al. 2020), and BD (Gu et al. 2023).

LncRNA MEG3 (Long noncoding maternally Expressed Gene 3) is widely considered a tumor suppressor because it is mostly downregulated in different cancer cell lines and primary human cancers like non-small cell lung cancer (Li et al. 2023), colorectal cancer (Elsherbeny et al. 2023), and osteosarcoma (Huang et al. 2023a, b).

On the other hand, lncRNA MAFG-AS1 (Long non-coding RNA musculoaponeurotic) is known to be an oncogene (Dabbaghi et al. 2023). It has been mentioned to be upregulated in multiple human cancers and commonly associated with bad prognosis (Ahmadi et al. 2023) extensive depth of invasion, and lymph node metastasis (Huang et al. 2023a, b). It was upregulated in prostate cancer (Li et al. 2022), and glioblastoma (Zhang and Li 2022).

MicroRNA 147-b has a putative participation in different diseases’ pathogenesis. In cancers, it is upregulated in some of them like tongue squamous cell carcinoma (Wong et al. 2008) and downregulated in others like human Colorectal Cancer (Yi et al. 2019). In myocardial infarction, its expression was low in the myocardium and its high level was thought to have a protective role by reducing both inflammation and apoptosis (Wu and Huang 2020). It was described to be induced inside macrophages and neutrophils upon TLR activation via stimulation of transcription factors like NF-KB in a negative feedback loop that works as a protective mechanism to alleviate the inflammatory response (Liu et al. 2009). Also, it is a precursor for both long noncoding RNA (lncRNA) MEG3 (Li et al. 2018) and lncRNA MAFG-AS1 (Cui et al. 2018).

Deluge research has been conducted on the role of both lncRNAs and miRNAs in BD pathogenesis and the possibility of using them as biomarkers for the disease and as therapeutic targets.

In the present study, we aimed to verify the diagnostic utility of lncRNA MEG3, lncRNA MAFG-AS1, and their precursor microRNA 147-b as novel biomarkers for BD.

## Materials and methods

### Study population

This study was performed in line with the principles of the Declaration of Helsinki. Approval was granted by the Ethics Committee of the Faculty of Medicine, Cairo University (Date 7/9/2022/No MD-218-2022).

A hundred participants were randomly chosen from the Internal Medicine Outpatient Clinic of the Faculty of Medicine, Kasr Al-Ainy Hospitals, Egypt. They were classified into 2 main groups.

- **Group 1:** 50 patients diagnosed with Behcet’s disease according to International Criteria for Behçet’s Disease (ICBD) criteria. There were 43 males and 7 females with a median age of 35 ranging from 18 to 50 years.
- **Group 2:** age and sex-matched 50 healthy controls (14 females and 36 males) with a median age of 37 ranging from 18 to 50 years.

### Diagnosis of Behcet’s disease and determining the activity of patients

We used ICBD criteria in diagnosing our BD patients (Davatchi et al. 2014). The Behçet’s Disease Current Activity Form (BDCAF) (Lawton 2004) and Behçet’s Syndrome Activity Score (BSAS) (Yilmaz et al. 2013) were our clinical tools to evaluate the activity of our patients.

Patients diagnosed by ICBD criteria and provided written informed consent were included in our study. Any patient diagnosed with malignancy, other autoimmune, auto-inflammatory, chronic inflammatory diseases, or diabetes were excluded. All patients and controls were assessed by history taking, and clinical examination to confirm diagnosis of BD. RNA extraction and measuring serum lncRNA MEG3, lncRNA MAFG-AS1, and miRNA 147-b of patients and controls by real-time PCR were done.

### Chemicals and equipment

Quantitative measurement of lncRNA MEG3, lncRNA MAFG-AS1, and miRNA 147-b was done by following the manufacturer’s instructions. miRNeasy mini kit was used to extract the total RNA, complementary DNA (cDNA) was produced by using miScript II reverse transcription kit, and quantitative polymerase chain reaction (qPCR) was done by miScript SYBR Green PCR kit (Qiagen, USA).

We have used a readymade miRNA 147-b primer (miRcury miRNA Assay has-miR-147b-5p), cat.no. YPO2119765. However, our lncRNAs primers were customized and their sequences are illustrated in Table 1. SNORD 68, Cat No. MS00033712 and GAPDH, cat.no. QT 300079247 were used as internal controls for miRNA and lncRNA respectively. NanoDrop spectrophotometer (NanoDrop Technologies, Inc., USA) was used for evaluating the quantification and purity of RNA, and Rotor-Gene thermocycler (Qiagen, USA) was used for qPCR programming.

**Table 1:** The primer sequences of lncRNA MEG3 (Wang et al. 2019), and lncRNA MAFG-AS1 (Tian et al. 2022)

Primer	Sequences
LncRNA MEG3	F: 5′CTGCCCATCTACACCTCACG-3′ R: 5′CTCTCCGCCGTCTGCGCTAGGGGCT-3′
LncRNA MAFG-AS1	F: 5′-ATGACGACCCCCAATAAAGGG-3′ R: 5′-CACCGACATGGTTACCAGC-3′

*F* forward, *R* reverse

### Calculation of results and statistical analysis

LncRNA MEG3, lncRNA MAFG-AS1, and miRNA 147-b expression levels were evaluated using the ΔCt method. Expression levels fold change was calculated by Equation 2^–ΔΔCt^. SPSS (Statistical Package for the Social Sciences) version 28 was our tool to analyze the results. Numbers and percentages were used to represent qualitative data. Quantitative variables were expressed as median (range). The Kolmogorov–Smirnov single-sample test was used to determine the data’s normality. The Mann–Whitney *U* test was used to compare two independent groups. Continuous data was correlated by using Spearman correlation. If the *p*-value equals 0.05 or less, it was considered significant. The diagnostic value of our 3 biomarkers was explored via receiver operation characteristics (ROC) curves and computation of the area under the curve (AUC).

## Results

### Activity manifestations and BDCAF and BSAS scores among the participants

**Fig. 1:**
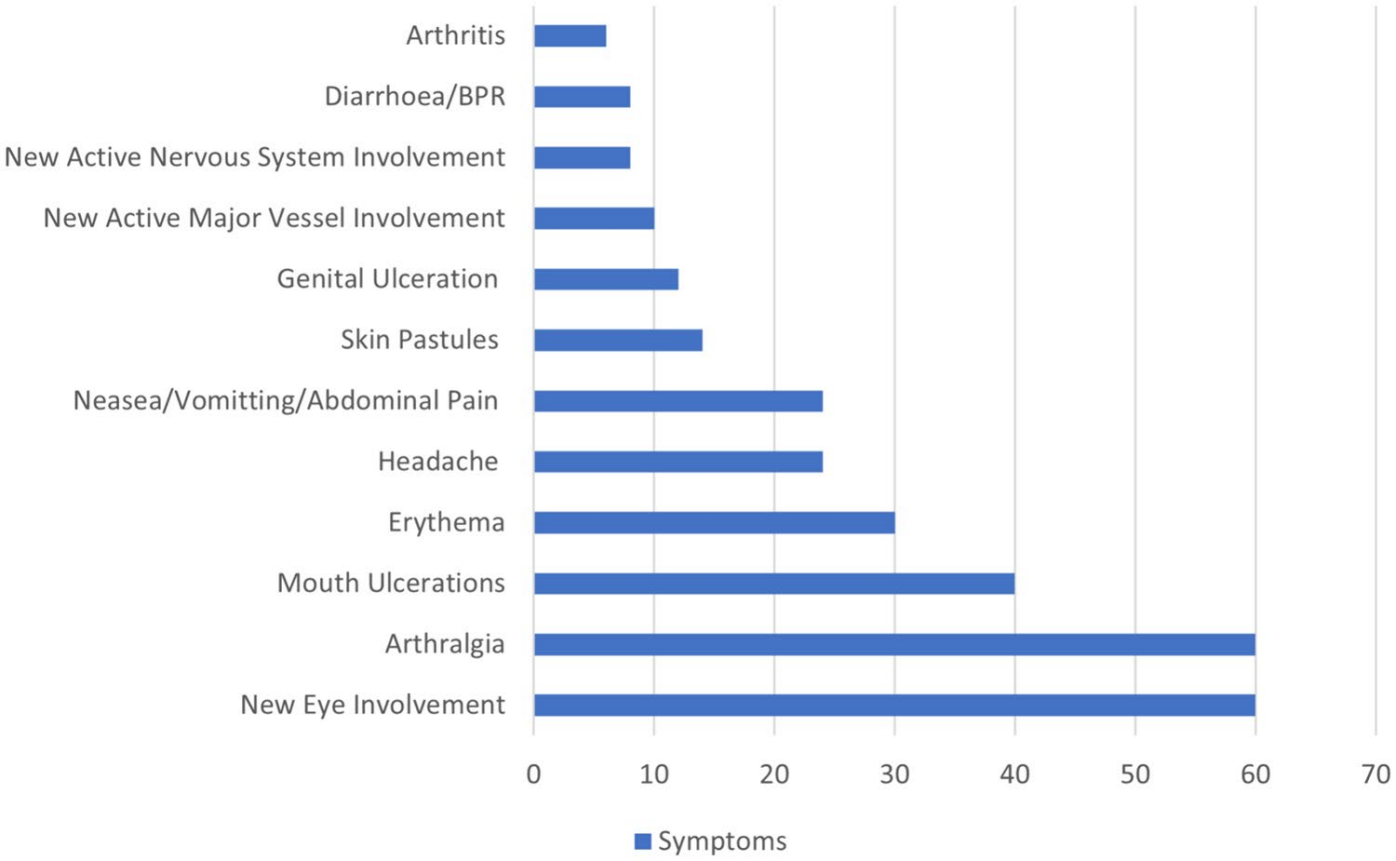
Activity manifestations among the participants in percentages. 60% of the patients had new eye involvement and joint arthralgia, followed by mouth ulcerations (40%) and erythema (30%) and the least is arthritis which was only present in 6% of the patients. This figure was done by using SPSS Statistics version 28

Different activity manifestations among the participants are illustrated in Fig. 1

### The expression levels and diagnostic accuracy of lncRNA MEG3, lncRNA MAFG-AS1, and miRNA 147-b in BD cases compared to the controls

**Fig. 2:**
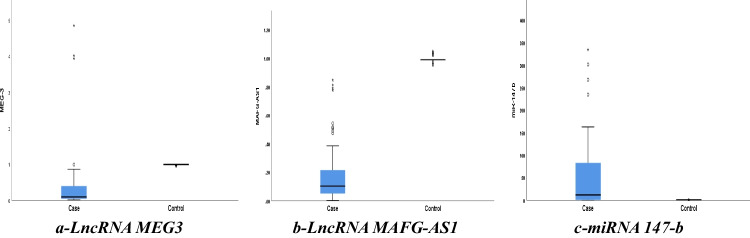
The gene expressions of lncRNA MEG3 (**a**), MAFG-AS1 (**b**), and miRNA 147-b (**c**) among the participants. A box plot represents data. The box represents the 25–75% percentiles; the median is represented by the line inside the box while the upper and lower lines represent the 10–90% percentiles. Points outside the 10th and 90th percentiles are stated by the filled points. This figure was done by using SPSS Statistics version 28

When lncRNA MEG3, lncRNA MAFG-AS1, and miRNA 147-b were assessed in the participants, both lncRNA MEG3 and lncRNA MAFG-AS1 were downregulated while miRNA 147-b was upregulated as seen in Fig. 2. The median MEG3 was 0.09, ranging from 0.01 to 4.8, the median MAFG-AS1 was 0.2, ranging from 0.001 to 0.9, and the median miRNA 147-b was 16.1, ranging from 0.1 to 334.3 with* p*-value <0.001 for all of them.

**Fig. 3:**
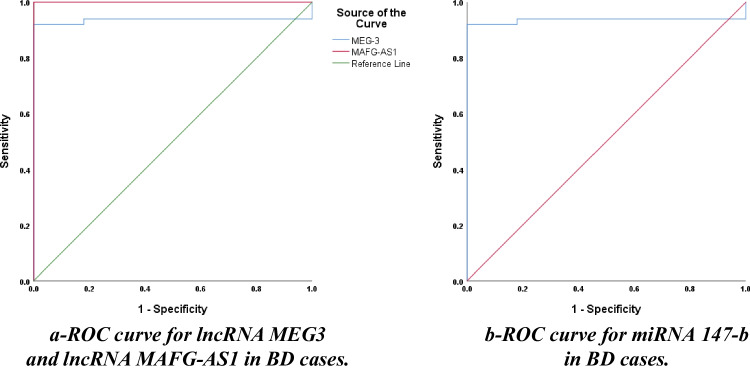
ROC curves **a** for lncRNA MEG3 and lncRNA MAFG-AS1 and **b** for miRNA 147-b in BD. This figure was done by using SPSS Statistics version 28

As seen in Fig. 3 and Table 2, the ROC curve for lncRNA MEG3 in BD shows AUC 0.936 with a cut-off value of 0.46, sensitivity of 76%, and specificity of 100%. The ROC curve for lncRNA MAFG-AS1 in BD has AUC 1 with a cut-off value of 0.899, sensitivity of 100%, and specificity of 100%. While miRNA 147-b ROC curve has AUC 0.78 with a cut-off value of 3.459, 70% sensitivity, and 100% specificity. The *p*-value was <0.001 for all of them.

**Table 2:** The diagnostic accuracy of lncRNA MEG3, lncRNA MAFG-AS1, and miRNA 147-b in detecting BD

Characteristics	Cut-off point	AUC	95% CI	Sensitivity	Specificity	*p*-value
MEG3	0.460	0.936	0.9–1	76%	100%	<0.001*
MAFG-AS1	0.899	1	1–1	100%	100%	<0.001*
miRNA 147-b	3.459	0.78	0.7–0.9	70%	100%	<0.001*

*AUC* area under the curve, *CI* confidence interval

*p value* < 0.05 (with asterisk"*") is statistically significant

### LncRNA MEG3 as a possible biomarker for predicting new eye involvement in BD

**Fig. 4:**
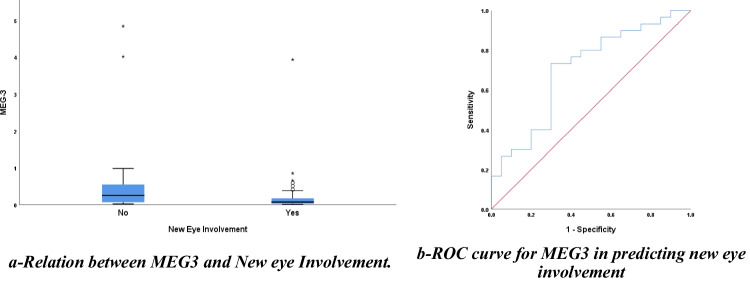
MEG3 in predicting new eye involvement. **a** A box plot represents data. The box represents the 25–75% percentiles; the median is represented by the line inside the box while the upper and lower lines represent the 10–90% percentiles. Points outside the 10th and 90th percentiles are stated by the filled points. **b** ROC curve for lncRNA MEG3 in detecting new ocular involvement. This figure was done by using SPSS Statistics version 28

Figure 4a shows a lower expression level of lncRNA MEG3 among patients who had new eye involvement in the last 4 weeks in comparison to those without new eye involvement (*p*-value=0.017). Figure 4b shows the ROC curve for MEG3 in predicting new eye involvement. Table 3 shows the diagnostic accuracy of MEG 3 in predicting new eye involvement.

**Table 3:** The diagnostic accuracy of lncRNA MEG3 in predicting new eye involvement in BD

Characteristics	Cut-off point	AUC	95% CI	Sensitivity	Specificity	*p*-value
MEG3	0.29	0.702	0.6-0.9	80%	55%	0.017*

*AUC* area under the curve, *CI* confidence interval

*p value* < 0.05 (with asterisk"*") is statistically significant

Table 3 shows the best cut-off point for MEG3 to predict new eye involvement was 0.09. The area under the curve was 0.702, 95% confidence interval (0.6–0.9), with a sensitivity of 55% and specificity of 71%.

## Discussion

BD is a chronic autoinflammatory disease, diagnosed clinically due to the absence of any pathognomonic test (Greco et al. 2018). This makes the patients suffer in a difficult journey from the onset of the disease till they know their diagnosis (Marinello et al. 2023). Therefore, major disabilities and a significant decrease in quality of life and work productivity are potential outcomes (Atas et al. 2019); this necessitates searching for tools to help in early diagnosis and proper management.

To sum up our results, BD patients revealed significantly downregulated lncRNA MEG3 and lncRNA MAFG-AS1, upregulated miRNA 147-b in comparison to controls. As well, those who had new BD ocular involvement in the previous 4 weeks showed significantly downregulated lncRNA MEG3 in comparison to those without new eye involvement.

Downregulated lncRNA MEG3 agrees with various previous studies including autoimmune and inflammatory diseases. For instance, in a study on periodontitis, lncRNA MEG3 was downregulated (Dong et al. 2020). Periodontitis is suggested to be a result of dysbiosis of the microbiota present inside the oral cavity. This can lead to the activation of several inflammatory mechanisms which may end in local and systemic autoimmune responses in different ways as TLR dysregulation (Suárez et al. 2020). Both BD and periodontitis are chronic inflammatory diseases that share common characteristics. Additionally, periodontal prophylaxis and treatment have a significant consequence in enhancing the quality of life in BD patients by balancing environmental triggers and found to have a relationship with the severity of BD (Aydındoğan et al. 2023).

Another study showed significantly downregulated lncRNA MEG3 in the serum of patients diagnosed with ankylosing spondylitis (AS). Notably, this was confirmed by induced AS in rats and providing MEG3 which inhibited AS progression (Liu et al. 2023). Besides, a study on rheumatoid arthritis described significantly low lncRNA MEG3 and this was augmented by its rising level during treatment (Wang et al. 2021). Furthermore, Multiple sclerosis, which is an autoimmune disease characterized by chronic inflammation of the central nervous system, revealed low expression levels of lncRNA MEG3 in relapsing phase patients in comparison to patients in remission and control groups (Torkamandi et al. 2021). In addition, the serum level of lncRNA MEG3 was significantly low in patients with sepsis, and they confirmed this relation by invitro culture of human macrophages which showed highly expressed MEG3 can reduce apoptosis of the macrophages and secretion of inflammatory factors via suppression of NF-KB signaling pathway (Pan and He 2020).

In contrast, our results are not in line with another study which showed that lncRNA MEG3 was significantly higher in BD patients in comparison to lupus patients and the control group (Mehana et al. 2022). Also, our results disagree with an animal study that described that lncRNA MEG3 aggravates chronic inflammation and insulin resistance in adipocytes by stimulating TLR4/NF-KB while its downregulation had the reverse actions (Lu et al. 2023). The different expression patterns between humans and rats can explain this.

Regarding the downregulated lncRNA MAFG-AS1, unlike cancer biology, MAFG-AS1 seems to act differently in inflammation. Intriguingly, it was also downregulated in periodontitis (Wangzhou et al. 2020). As mentioned before periodontitis and BD are related to each other’s. Otherwise, most of the other studies that investigated lncRNA MAFG-AS1 showed upregulation of its level in a limited number of infections and different types of cancers. For instance, it was upregulated in hepatitis B virus-infected liver tissues (Zhang et al. 2021), in dengue infection (Zhang et al. 2023), and in breast cancer (Gao et al. 2022).

As well, our study also shows an upregulation of miRNA147-b in BD cases in comparison to controls, which is in coherence with previous studies that showed that there was a high level of miRNA147-b in the serum and colonic mucosa of a dog with intestinal inflammatory bowel disease (Konstantinidis et al. 2020), and in liver biopsies related to patients having chronic hepatitis C infection (Lin and Hu 2021). Also, a former study referred to it as an immune response-related miRNA that increased upon treating human monocytes with insect-derived peptides in a trial to suppress Dengue viruses (Limthongkul et al. 2019). Its high expression may be explained to decrease excessive inflammatory response as mentioned before (Liu et al. 2009). This opinion agrees with a study stating it as an anti-inflammatory RNA that may be used to alleviate inflammation via suppressing the TLR4/NF-KB signaling pathway in macrophage and they recommended it to be used in treating tendon injuries (Shen and Lane 2023). On the other hand, our results disagree with a study that described miRNA147-b to be significantly downregulated in colons of rats with experimental Crohn’s disease (Wei et al. 2015); this difference may be rationalized by different timing of miRNA evaluation in the course of the disease (Lin and Hu 2021).

Additionally, clinical data of our study shows that 60% of our patients had new ocular involvement and this was associated with a significantly low expression of lncRNA MEG3; this can be an extension of the inflammatory status of the body to the eye. A previous study suggested that a low level of lncRNA MEG3 participates in ocular angiogenesis in diabetic retinopathy (Zhang et al. 2019), and another study has mentioned neovascularisation as one of the most common features in BD patients (Posarelli et al. 2020). Another study confirmed this by injection of MEG3 on a virus intravitreal to result in improvement of retinal angiogenesis (Sharma and Singh 2023). These findings suggest that lncRNA MEG3 is a possible novel biomarker that may participate in the pathogenesis of ocular affection and can be investigated thoroughly to confirm the possibility of using it in predicting new ocular BD involvement.

**Fig. 5:**
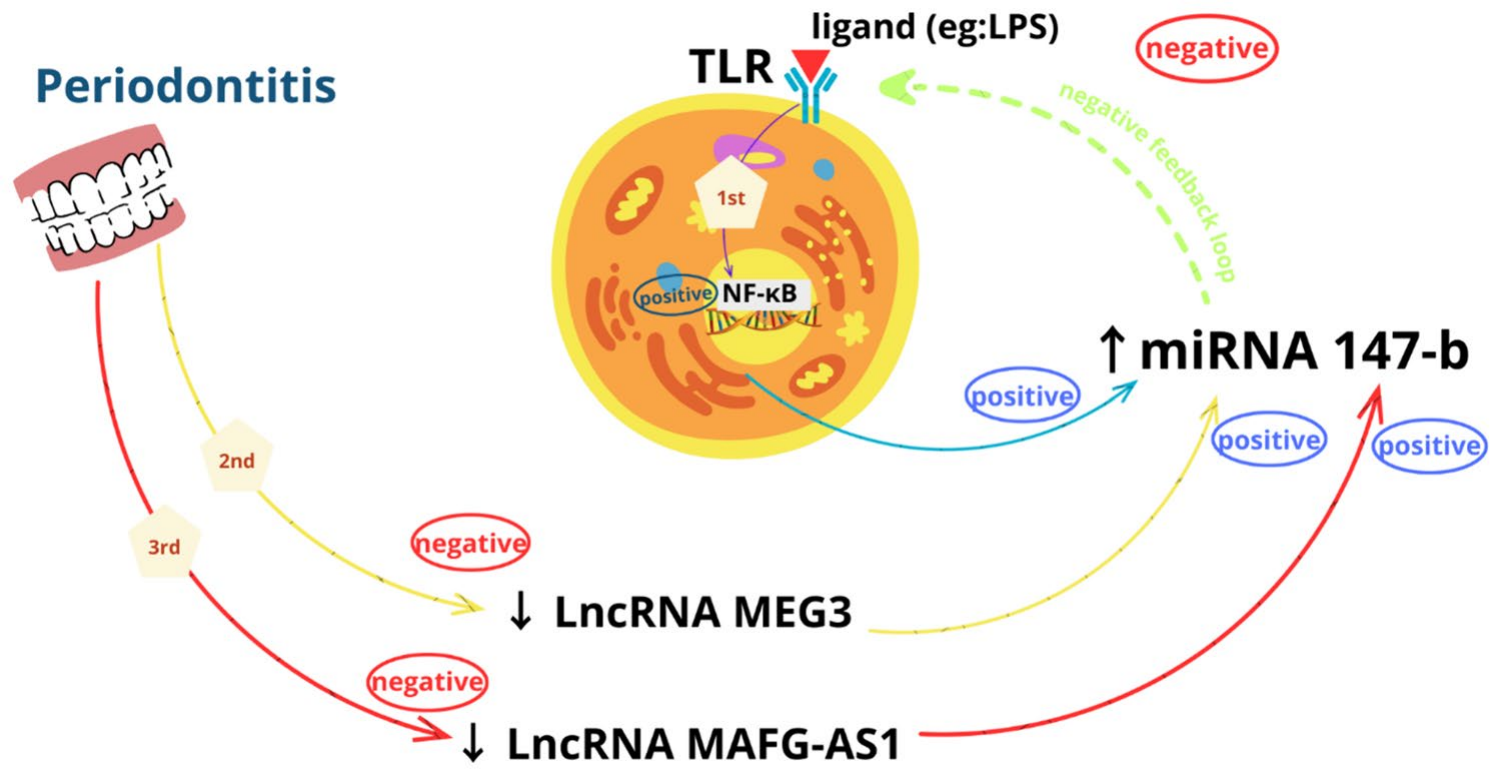
This graph shows the three possible mechanisms for BD suggested by the present study. The first one is started by stimulation of TLRs by lipopolysaccharides. This leads to the stimulation of NF-KB which will cause an increase in miRNA 147-b. MiRNA 147-b will inhibit TLRs in a negative feedback loop to alleviate the inflammation (the green line). Periodontitis is the start for both second and third ones (the yellow and red lines), it is associated with downregulation of both lncRNA MEG3 and lncRNA MAFG-AS1. Their downregulation will increase miRNA 147-b even directly by not sponging it or through other mediators. The resulting high miRNA 147-b will end also in the suppression of TLRs as mentioned before to suppress the inflammation. This figure was done by using the Canva program

Lastly, we want to clarify our points in this study, we are pointing to three possible mechanisms for BD as seen in Fig. 5. They may be more intercorrelated with each other, but this needs more research to elucidate the detailed genetic pathways. The first axis is through the stimulation of TLRs by a ligand, e.g., LPS (lipopolysaccharides), which stimulates NF-KB. Then, NFKB will increase miRNA 147-b, which will inhibit TLRs in a negative feedback loop to alleviate the inflammation (Liu et al. 2009). The second and the third ones are through periodontitis. As mentioned above, periodontitis was associated with the downregulation of both lncRNA MEG3 and lncRNA MAFG-AS1. Their downregulation will increase miRNA 147-b even directly by not sponging it or through other mediators. The resulting high miRNA will end also in the suppression of TLRs as mentioned before to suppress the inflammation.

## Conclusion

We are introducing lncRNA MEG3, lncRNA MAFG-AS1, and their precursor miRNA 147-b as potential promising diagnostic markers and therapeutic targets for BD. LncRNA MEG3 can be used as a potential novel biomarker in predicting new ocular BD involvement. Future research should be conducted to study their downstream target genes and signaling pathways.

## Data Availability

All data are available upon reasonable request from the corresponding author.
